# Ventromedial Prefrontal Cortex Activity and Sympathetic Allostasis During Value-Based Ambivalence

**DOI:** 10.3389/fnbeh.2021.615796

**Published:** 2021-02-22

**Authors:** Neil M. Dundon, Allison D. Shapiro, Viktoriya Babenko, Gold N. Okafor, Scott T. Grafton

**Affiliations:** ^1^Department of Psychological & Brain Sciences, University of California, Santa Barbara, Santa Barbara, CA, United States; ^2^Department of Child and Adolescent Psychiatry, Psychotherapy and Psychosomatics, University of Freiburg, Freiburg im Breisgau, Germany; ^3^Department of Psychology, University of California, Berkeley, Berkeley, CA, United States

**Keywords:** approach-avoidance, sympathetic stress, ventromedial prefrontal cortex, pre-ejection period, ambivalence, value-based decision making

## Abstract

Anxiety is characterized by low confidence in daily decisions, coupled with high levels of phenomenological stress. Ventromedial prefrontal cortex (vmPFC) plays an integral role in maladaptive anxious behaviors via decreased sensitivity to threatening vs. non-threatening stimuli (fear generalization). vmPFC is also a key node in approach-avoidance decision making requiring two-dimensional integration of rewards and costs. More recently, vmPFC has been implicated as a key cortical input to the sympathetic branch of the autonomic nervous system. However, little is known about the role of this brain region in mediating rapid stress responses elicited by changes in confidence during decision making. We used an approach-avoidance task to examine the relationship between sympathetically mediated cardiac stress responses, vmPFC activity and choice behavior over long and short time-scales. To do this, we collected concurrent fMRI, EKG and impedance cardiography recordings of sympathetic drive while participants made approach-avoidance decisions about monetary rewards paired with painful electric shock stimuli. We observe first that increased sympathetic drive (shorter pre-ejection period) in states lasting minutes are associated with choices involving reduced decision ambivalence. Thus, on this slow time scale, sympathetic drive serves as a proxy for “mobilization” whereby participants are more likely to show consistent value-action mapping. In parallel, imaging analyses reveal that on shorter time scales (estimated with a trial-to-trial GLM), increased vmPFC activity, particularly during low-ambivalence decisions, is associated with decreased sympathetic state. Our findings support a role of sympathetic drive in resolving decision ambivalence across long time horizons and suggest a potential role of vmPFC in modulating this response on a moment-to-moment basis.

## Introduction

Decisions can be so easy; approaching a large reward with minimal associated costs or avoiding a small reward with excessive associated costs requires precious little deliberation and minimal supportive decisional architecture. Unfortunately, many real-world goal-directed decisions impart “ambivalence,” i.e., a more ponderous appraisal of marginally valent subjective values born out of near-equivalent underlying reward and cost. Accordingly, value-based decisions modulate brain activity across a wide network of orbitofrontal, lateral and medial prefrontal, and anterior cingulate cortices (Rangel et al., 2008; Basten et al., 2010; Amemori and Graybiel, 2012; Levy and Glimcher, 2012; Talmi and Pine, 2012; Bartra et al., 2013). In particular, ventromedial prefrontal cortex (vmPFC) is linked to at least two value-based decision parameters: coding for subjective value (high or low likelihood of approach) and ambivalence (proximity to the approach/avoid threshold; Rolls et al., 2010; De Martino et al., 2013; Lebreton et al., 2015). Recent human imaging data has further parcellated vmPFC's dual value-based functionality into temporally contingent roles, i.e., coding for subjective value during offer appraisal, and then for confidence, the inverse of ambivalence, during choice commitment (Shapiro and Grafton, 2020).

Optimizing the net yield from marginal value-based decisions in ever-changing environments presents an evolutionary challenge for any organism that must approach or avoid opportunities for resources. Consistent value-action mapping should guide an organism to always approach opportunities that promise a net-positive value and avoid those likely to hold a net-negative value, even when the valence is only marginal. However, there are physical constraints that must be overcome, all while maximizing physiological efficiency. Resolution of value-based ambivalence is therefore highly likely to interact with anticipatory allostatic control (Sterling and Eyer, 1988), a process observed across mammals where the brain makes predictive adjustments to physiological systems, such as metabolism, blood pressure, heart-rate and core temperature, preemptively increasing the likelihood of equilibrium between external demands and the internal milieu (Schulkin and Sterling, 2019). Efficient and predictive allostatic control reduces the need for delayed feedback-based homeostatic error correction, leading to greater evolutionary fitness (Schulkin and Sterling, 2019). Interestingly, separate branches of non-human and human research have implicated vmPFC in allostatic control, given first its top-down influence on the adrenal medulla (Dum et al., 2016, 2019), and also its neural projections to other sites within an integrated visceromotor-interoceptive system, including cingulate, dorsal amygdala and various sites along the insula (Kleckner et al., 2017).

Whether value-based ambivalence drives allostasis, and what role, if any, is played there-in by vmPFC are outstanding questions that are considered in the present research. Notably, the combination of cognitive, physiological and neural deficits that present in anxiety disorders inspired a hypothesis regarding behavior-sympathetic-neural relations that might characterize value-based ambivalence allostasis. First, people experiencing anxiety are particularly challenged by decision ambivalence. One of the core cognitive deficiencies of anxiety disorders is a pervasive bias toward negative or aversive appraisals of neutral or ambiguous situations (Clark and Wells, 1995; Hirsch and Mathews, 1997; Constans et al., 1999). More recently, data from approach-avoidance tasks specifically identify ambiguity (and not aversiveness) as a key attenuator of approach behavior in anxiety groups (Kuckertz et al., 2017), while computational models of choice reaction time have further suggested that negative biases in anxiety are not reflexive, but rather that they arise as a result of wayward Markovian decision formation. That is, an accumulation of evidence that begins equidistant from approach and avoid decision criteria, but with gradients that default toward an aversive judgment (Aylward et al., 2020).

Second, anxiety is closely associated with increased autonomic mediated stress and recent studies have identified specific anxiety deficits in the sympathetic branch of the stress system (Shields and Slavich, 2017). This association is consistently observed at different stages of the lifespan (Funke et al., 2017; Fu et al., 2018) and supported further by links between pervasive anxiety and increased risk of cardiac ischemia, myocardial infarction and sudden cardiac death (Samuels, 2007; Frasure-Smith and Lespérance, 2008; Martens et al., 2010; Fisher and Newman, 2013). Importantly, there also appears to be a link between anxiety-related decision biases and sympathetic dysregulation. For example, compared to controls, anxious youths who show negative biases in decision tasks show elevated sympathetic states, measured by electrodermal assays, during a Trier task (Rozenman et al., 2017). These findings are consistent with behavior known to reduce anxiety–such as conscious regulation of respiratory cadence–reducing the sympathetic dominance of autonomic output (Jerath et al., 2015), and more generally, with the anxiolytic effects of exercise (Petruzzello et al., 1991).

Third, vmPFC demonstrates specific functional differences in anxiety. Recent human neuropsychological evidence, using fear-generalization paradigms with Generalized Anxiety Disorder patients (GAD), shows decreased vmPFC activations while patients evaluated “safe” stimuli, i.e., those that had not been conditioned with an aversive outcome (Greenberg et al., 2013; Via et al., 2018). These findings suggest pervasive anxiety may stem from a continuous anticipation of harm, a response which is otherwise attenuated by vmPFC in healthy controls. These findings also serve as a useful predictive model for vmPFC's functional role within an allostatic response to decision ambivalence, namely, mediating sympathetic state so as to only increase when situations most require it, or conversely, to decrease sympathetic output when ambivalence is low.

**Figure 1:**
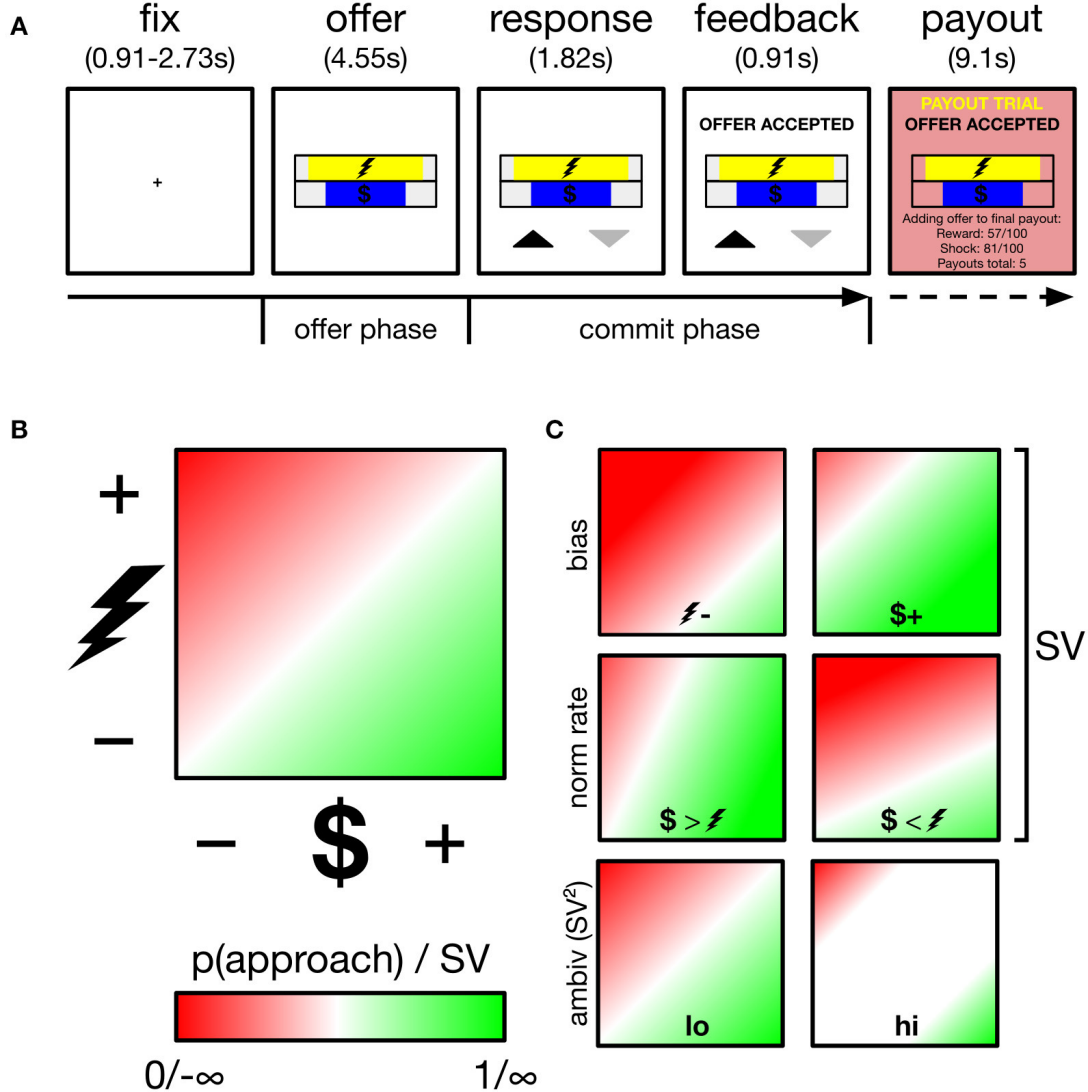
Overview of task, decision space and schematics of subjective value (*SV*) parameters and ambivalence. **(A)** Participants performed an approach-avoidance task, trading off varying levels of monetary reward for varying levels of painful electric shock, respectively, depicted by the length of different colored bars. Participants appraised offers during the offer phase, and committed their response during the commit phase, indicated by appearance of response mapping (upward arrow–approach). Low probability (~5%) payout trials were added to a cache to be delivered outside the scanner. **(B)** Trials drawn from two-dimensional decision space; reward axis ranged from $0.01 to $1.50, cost axis ranged from mild discomfort to near unbearable pain (individually calibrated). p(approach) ranging from 0 to 1 corresponds to *SV* ranging from −∞ to +∞. The example gradient depicts zero baseline bias (i.e., no minimum reward required to approach any cost, no minimum cost tolerated to approach any reward) and equal normalization rate of reward and cost (1 unit reward = 1 unit cost). *SV* is a function of these two parameters. White region along the diagonal reflects maximal ambivalence (p(approach)=0.50; *SV*=0). **(C)** Example gradients resulting from varying the baseline bias (top), normalization rate (middle) or ambivalence (bottom).

Taken together, these three branches of the anxiety literature suggest that impairments with ambiguous or ambivalent decisions might potentially be linked to sympathetic dysregulation, and that vmPFC could be an important node in regulating the sympathetic response. An important next step is to characterize the relationship between sympathetic state and decision ambivalence in neurotypical people. Sympathetic state could simply be aligned with increased ambivalence, in line with a homeostatic response to the phenomenology of difficult decisions. However, we recently demonstrated a more nuanced and adaptive contribution of sympathetic drive, with heightened responses associated with optimal decisions, but only in an environment of deteriorating resources (Dundon et al., 2020). Alternative sympathetic associations also need to be considered. For example, when faced with a compound offer of reward and cost, sympathetic state might simply track either or both objective levels of immediately available reward or cost; e.g., if a generic reward or threat appears, sympathetic state might elevate during an approach or avoid. Such a response would be response-driven and homeostatic, i.e., error-corrective. Sympathetic state may also track subjective value (*SV*), i.e., a subjective measure that integrates impending reward and cost into a net yield, which is filtered through individualized parameters that account for baseline biases (e.g., is a certain reward needed to approach any cost) and normalization rates (e.g., how much is each unit of reward worth in units of cost; see Figures 1B,C).

To test these alternative links between sympathetic mediated allostasis, decision making in an approach-avoidance task, and the role of the vmPFC, we capitalize on a promising assay of cardiac autonomic physiology to characterize sympathetic beta-adrenergic myocardial mobilization (Richter et al., 2016; Cieslak et al., 2018). Combined, the electrocardiogram (EKG) and impedance cardiogram (ICG) can identify the pre-ejection period (PEP) of individual heartbeats, and evaluate sympathetic state modulations (shorter PEP, higher/mobilized sympathetic state, and vice versa) across time horizons as short as a few seconds (Kuipers et al., 2017). This approach has already charted sympathetic modulations by cognitive parameters such as reward relevance (Wright et al., 2002; Richter and Gendolla, 2009), task difficulty (Richter et al., 2008; Kuipers et al., 2017), deterioration of environmental richness (Dundon et al., 2020) and stimulus aversiveness (van Hedger et al., 2017; Ogden et al., 2019). State of the art pre-processing techniques also allow impedance cardiogram equipment to reliably capture fluctuations in sympathetic state while human participants are in a functional magnetic resonance imaging environment (fMRI; Cieslak et al., 2018).

In the present study we accordingly combine EKG/ICG with fMRI, while healthy human participants perform a value-based approach-avoidance task. Our study addresses first the outstanding behavior-sympathetic question: how does sympathetic drive fluctuate during approach-avoidance decision making. We demonstrate that sustained periods of increased sympathetic state (lasting minutes) aligns with periods of more consistent value-action mapping across multiple decision-making trials. We then address the outstanding behavior-sympathetic-neural question to assess the correspondence between choice behavior, sympathetic drive and activity in vmPFC. The results show that we can extend a role of vmPFC to sympathetic regulation of ambivalence that is parsimonious with a resource model. Namely, as vmPFC activation increases, sympathetic drive (PEP) decreases. However, this association only emerges when participants are faced with low-ambivalence offers and decide to avoid them. This countercyclical relationship between vmPFC and sympathetic state in scenarios of low ambivalence avoidance aligns the region's role in sympathetic allostatic control with prior studies linking its inhibitory response with fear generalization. We combine these behavioral, cardiac and neural findings to propose that value-driven ambivalence elicits an adaptive allostatic sympathetic response, which is tuned by response-specific gain control in vmPFC.

## Materials and Methods

### Subjects and Experimental Overview

Subjects were a subset from a larger fMRI study (*n* = 28) of approach-avoidance decision making (Shapiro and Grafton, 2020). We present data from all cases in that study who underwent fMRI with simultaneous EKG/ICG (*n* = 24). Two participants were removed from analyses during preprocessing, respectively, due to excessive noise in their impedance cardiogram and MRI (susceptibility artifacts). We accordingly report data from a final group of 22 participants. This group's mean (standard deviation) age at time of testing was 21.4 (2.86). Fifteen participants were women, and 20 were right-handed. Participants reported no history of neurological injury and no use of psychoactive medication at time of testing. Participants provided informed written consent to participate in the experiment, which was conducted on a single day. The task, electric shock procedure, payout structure and neuroimaging protocol were all identical to Shapiro and Grafton (2020). Participants were recruited via both word-of-mouth and an online participant recruitment portal, and all procedures were approved by the Institutional Review Board at The University of California, Santa Barbara.

### Task

Subjects performed an approach-avoidance task, in which they approached or avoided offers of money (reward) paired with electric shock (cost). Each trial drew a different deterministic offer from a two-dimensional decision space (see Figure 1B). The horizontal length of two horizontal bars, centrally positioned one on top of the other, indicated the magnitude of reward ($0.01 to $1.50) and cost (minimum to near-max pain tolerance–see thresholding procedure below) offered on a given trial. Participants either approached both the trial's reward and cost, or avoided both. Across participants, the reward bar was either always yellow or always blue (cost bar vice versa). To minimize learning and memory requirements, the reward bar was overlaid with a $ symbol, while the cost bar was overlaid with the symbol of a lightning bolt. The relative position of each bar (i.e., top or bottom) alternated across runs. Throughout the experiment, participants registered choices using the index and middle finger of their right hand, removing potential choice biases arising from previous hand usage (Valyear et al., 2019), and reducing neural signal generation from effector selection substrates in motor and parietal regions (Fitzpatrick et al., 2019). The mapping of choice (approach, avoid) onto finger (index, middle) also varied trial-by-trial, to minimize both habitual responding and the contamination of decisional neural signals by action preparation signals (see trial structure below).

*Trial structure* is depicted in Figure 1A. Each trial began with a central fixation cross lasting either 910, 1,820, or 2,730 ms. Offer stimuli then appeared for 4,550 ms (referred to as the *offer* phase in later analyses). Participants were instructed to use this phase to evaluate the offer and that responses could not yet be registered. After the offer phase, response mappings appeared below the offer stimuli for a further 1,820 ms, informing participants both of that trial's mapping and that they could now register a response. Mapping was indicated by an upward (approach) and downward (avoid) pointing arrow. Both arrows appeared equidistant from the screen center and its lower periphery, with one to the left and one to the right of the central meridian, and both equidistant from the meridian and its respective vertical periphery. The spatial mapping of arrow position to finger response was fixed across all trials and participants: (left–index, right-middle). Despite this enforced delay before registering responses, RTs showed modulation by ambivalence (see Supplementary Materials: *Behavioral alignment between original and current sample* and panel B of Supplementary Figure 1) verifying that decisions were not wholly formed during the offer period of trials. Participants registered responses on a Lumina LSC-400 controller and could change their choice for the duration of the response phase. Following the response phase, verbal feedback appeared for 910 ms above the offer stimuli and instructed the participant of their final choice. Together, the response and feedback phases comprise the commit phase in later analyses. On payout trials (see payout trials below), additional verbal feedback appeared for 9,100 ms (payout phase), instructing participants that the reward and cost of the current trial was either added to their payout cache (if approached) or not (if avoided). Payout feedback also presented a running tally of how many payouts were in the cache. Participants performed 189 trials in total, divided into six runs of either 31 or 32 trials. Runs lasted ~5 min.

*Payout trials* were used in lieu of administering electric shocks while participants were in the fMRI. Ten of each subject's trials were pseudorandomly selected as payout trials. As described above, these trials provided additional feedback that their reward and cost would be received after the scanning session, if that trial had been approached. Payout trials were only revealed following the commitment of a response and participants were requested to treat every trial as a potential payout trial. Payout trial selection was also constrained to never include trials above 80% of their maximum pain tolerance (see below), though this was not disclosed to participants (Note that payout trials did not affect behavior on the immediate following trial; see Supplementary Materials: *Control gLME for effects of payout awareness)*.

*Thresholding procedure:* the cost dimension of each subject's decision space was calibrated to cover a range of pain from their subjective minimum to a near-maximum level, i.e., controlling for individual differences in pain tolerance. Cutaneous electrical shocks (1s duration; *f* = 100Hz; λ = 2ms) were applied via two adhesive electrodes applied to the back of the hand, administered using a constant current stimulator and controlled by a train generator (respectively, models DS7A and DG2A, Digitimer, Great Britain). Using this setup, pain is modulated via voltage. Beginning at 1 mV, and at gradually increasing voltages, participants reported (1) the minimal voltage where they perceived the shock, (2) the minimal voltage that began to cause discomfort, and finally (3) the voltage that caused the pain to become unbearable. When participants first declared that the voltage level corresponded to unbearable pain, experimenters would follow up by asking them to confirm that this was the maximum pain they could possibly withstand, which usually led to participants accepting a further increase in voltage. Once participants confirmed they had reached an unbearable level of pain, they received a sample of 14 shock intensities between (2) and (3) (intensities not verbally disclosed), and they reported the level of pain on a rating scale of 0–10. This procedure was repeated twice to account for habituation. The cost dimension of each subject's decision space ranged from the second estimate of (2) above, to the second estimate of (3) above. Participants were familiarized to this range of cost with a sample of shocks, corresponding to 0.05, 0.25, 0.50, 0.75, and 0.95 of this scale. A sigmoid fitted to the second set of ratings (as a function of voltage) determined the voltage associated with a subject's 80% maximum level of pain tolerance (80 max). Unbeknownst to the participant, trials with a cost >80 max were excluded from the pseudorandomly selected set of payout trials.

### Cardiovascular Physiology Protocol

EKG and ICG data were recorded using ten EL500 electrodes, bridged to the skin with BIOPAC GEL100 (BIOPAC, USA). Two EKG electrode locations were, respectively, beneath the right collarbone and left rib cage. Eight ICG electrode locations were from Bernstein (1986): two on each side of the neck and two on each side of the torso. Each location was cleaned with an abrasive pad and exfoliated with NuPrep gel (ELPREP, BIOPAC, USA) prior to electrode attachment. ICG electrodes provided the ground. EKG and ICG were, respectively, recorded using an ECG100C and NICO100C amplifier (both from BIOPAC, USA) and integrated using an MP150 system (BIOPAC, USA). EKG and ICG data were recorded at 5 kHz. Online, the raw (z) ICG data were differentiated with respect to time (dz/dt) and then high-pass filtered to remove respiratory artifact. Online processing and data storage was managed with AcqKnowledge software version 4.3 (BIOPAC, USA).

### Estimates of Sympathetic State–PEP

Assays of sympathetic state at each heartbeat (k) were the pre-ejection period (PEP(k)), i.e., the time between initial innervation of the left ventricle (corresponding to the R point of the QRS complex of the EKG) and the opening of the aortic valve (corresponding to the B point of the ICG's dz/dt). B point identification was augmented using the semi-automated moving ensemble average pipeline (MEAP; Cieslak et al., 2018). Shorter PEP values reflect increased sympathetic cardiovascular drive. For statistical analysis sympathetic state was summarized across two time horizons–(1) trial-by-trial estimates were mean PEP values across each heartbeat that occurred during the offer phase (offer PEP) of each trial and the period spanning choice and feedback (but never payout) phases (commit PEP) on each trial. (2) run-based estimates were the median of collapsed offer PEP and commit PEP values across each trial of a single run, i.e., gauging a summary of the sympathetic state related to decisional activity on a run-by-run basis.

### Behavior-Sympathetic Analyses

#### Modeling Overview

We used linear mixed effects models to explore the relationship between sympathetic state and variables related to choices across two time horizons: (1) on a trial-by-trial basis (trial-by-trial models), i.e., associations between the sympathetic state on a certain trial and the characteristics of that trial's choice and (2) on a summary basis (summary models), i.e., the summary of the sympathetic state across a longer time horizon (a run of ~30 trials, lasting several minutes) and summaries of the choice characteristics across all trials on that run. In each case, models were fitted using the lme4 package for R (Bates et al., 2015) with restricted maximum likelihood approximation, and summary statistics were assessed with both the lme4 and lmerTest (Kuznetsova et al., 2017) packages in R. Each model fitted a fixed effect for each specified coefficient, and an individual intercept for each subject (random intercept model). Both classes of models also contained a continuous regressor to account for trial number and a categorical nuisance regressor to account for variance ascribed to the order of a given run in the experiment (1 to 6). All continuous regressors were scaled across subjects and log-transformed if positively skewed. Note that the primary difference between the trial-by-trial and summary models therefore relates to the number of rows per subject: trial-by-trial models had as many rows as trials, while summary models contained six rows relating to each of the experimental runs.

#### Estimating Subjective Value and Ambivalence, Trial-by-Trial

Value reflects the net yield from the objective reward and cost of an offer. Reward exceeding cost presents a positive value, and vice versa. However, reward and cost might be weighted differently across individuals; reward seekers will approach proportionately more cost, while cost avoiders will avoid proportionately more reward. To account for such individual differences, we used logistic models to estimate the magnitude of *SV* available on each trial. Separately for each subject, trialwise choice probability *p(approach)* was modeled as a function of an intercept, the magnitude of reward and the magnitude of cost offered on each trial of the task, using a logistic transfer function. *SV* on trial (*t)* can therefore be computed with:

(1) $$\begin{array}{l} SV\left(t\right)=log\left(\hat{p}\left(t\right)\;/1-\hat{p}\left(t\right)\right) \end{array}$$

Where $\hat{p}$ is the modeled probability of approaching the offer on trial *(t)* from:

(2) $$\hat{p}(t)=e^{\hat{y}(t)}\;/\;(e^{\hat{y}(t)}+1)$$

(3) $$\begin{array}{l} \hat{y}\left(t\right)=\beta^{⊤}X\left(t\right) \end{array}$$

Where three-element column vector *X*(*t*) contains a constant term (1) and the reward and cost offered on trial *(t)*, and β contains the parameters estimated across all trials using maximum likelihood. *SV* ranges from negative infinity (low *SV*; readily avoided offers) to positive infinity (high *SV*; readily approached).

Figure 1C demonstrates how two underlying parameters determine *SV*: baseline bias and normalization rate. To estimate these parameters, model-estimated $\hat{p}$ values are first computed across the entire theoretical decision space. A linear function then finds the best coordinates of the approach/avoid threshold, i.e., where $\hat{p}$ = 0.50 (corresponding to *SV*=0). This threshold is characterized by an *intercept* term and a *slope*. *Intercept* is the baseline bias, which reflects the degree to which a subject requires a fixed level of reward before any magnitude of cost is approached, or conversely if a subject tolerates a fixed level of cost to obtain any level of reward. Mapping reward onto the x-axis, as we do in our analyses, these biases are, respectively, demonstrated with an *intercept* below and above 0. *Slope* is the normalization rate, which determines whether participants value single units of reward more than single units of cost (or vice versa). In our analyses, a more positive slope reflects a greater weighting of reward units vs. cost, and vice versa.

Ambivalence arises when an offer falls close to an individual's approach/avoid threshold, i.e., where *SV*=0, the offer is equally likely to be approached and avoided (high ambivalence). Where *SV* approaches ±∞, the offer is instead low ambivalence, and will be either always approached (+∞) or always avoided (−∞). Accordingly, *ambivalence* = *SV*^2^ yields a measure of ambivalence, with values ranging from 0 (high ambivalence) to ∞ (low ambivalence).

### Estimating Subjective Value and Ambivalence, Summarized by Run

As specified above, we fitted two classes of models, relating to short and long time horizons. The above procedures relate to *SV* and *ambivalence* computation for single trial models (short time horizon). To summarize decision parameters for specific runs (long time horizon), we fitted the logistic regression model above separately for each run of 31 or 32 trials. The run-specific logistic models allowed us to compute run-specific estimates of the baseline bias and normalization rate, which would not be possible from a model across all trials. The resulting run-specific vector of parameters was then used to estimate *SV* for each trial of a given run, and *ambivalence* was again computed with *SV*^2^. We, respectively, used (i) median *SV*, (ii) median *ambivalence*, (iii) *intercept*, and (iv) *slope*, to summarize the subjective value, ambivalence, baseline bias and normalization rate for the trials of a given run *(r)*.

### Objective Reward and Cost Models

As a control measure to ensure sympathetic state was modulated by subjective and not objective levels of reward and cost, we ran two additional control models (one trial-by-trial, one summary) which tested the association between sympathetic state and the objective level of reward and cost (either trial-by-trial or summarized). In these models we also included a proxy for objective ambivalence–the product of normalized reward and cost.

### Behavior-Sympathetic Linear Mixed Effects Model Synopsis

We fitted a total of eight linear mixed effects models across our trial-by-trial (1A-1B) and summary (2A-2F) classes:

$$\begin{array}{l} symp\;state\left(t\right)=z_{1A}\left(n\right)+{\beta 1}_{1A}*reward\left(t\right)+{\beta 2}_{1A}*cost\left(t\right)\\ +{\beta 3}_{1A}*reward{\left(t\right)}^{*}cost\left(t\right)+{\beta 4}_{1A}*run\left(t\right)+{\beta 5}_{1A}*trial\left(t\right)+\epsilon_{1A}\left(t\right)\\ \left[model\;1A\right]\\ symp\;state\left(t\right)=z_{1B}\left(n\right)+{\beta 1}_{1B}*ambivalence\left(t\right)+{\beta 2}_{1B}*SV\left(t\right)\\ +{\beta 3}_{1B}*run\left(t\right)+{\beta 4}_{1B}*trial\left(t\right)+\epsilon_{1B}\left(t\right)\\ \left[model\;1B\right]\\ symp\;state\left(r\right)=z_{2A}\left(n\right)+{\beta 1}_{2A}*reward\left(r\right)+{\beta 2}_{2A}*cost\left(r\right)\\ +{\beta 3}_{2A}*run\left(r\right)+\epsilon_{2A}\left(r\right)\\ \left[model\;2A\right]\\ symp\;state\left(r\right)=z_{2A}\left(n\right)+{\beta 1}_{2A}*ambivalence\left(r\right)+{\beta 2}_{2A}*SV\left(r\right)\\ +{\beta 3}_{2A}*run\left(r\right)+\epsilon_{2A}\left(r\right)\\ \left[model\;2B\right]\\ offer\;symp\;state\left(r\right)=z_{2B}\left(n\right)+{\beta 1}_{2B}*ambivalence\left(r\right)\\ +{\beta 2}_{2B}*SV\left(r\right)+{\beta 3}_{2B}*run\left(r\right)+\epsilon_{2B}\left(r\right)\\ \left[model\;2C\right]\\ commit\;symp\;state\left(r\right)=z_{2C}\left(n\right)+{\beta 1}_{2C}*ambivalence\left(r\right)\\ +{\beta 1}_{2C}*SV\left(r\right)+{\beta 1}_{2C}*run\left(r\right)+\epsilon_{2C}\left(r\right)\\ \left[model\;2D\right]\\ symp\;state\left(r\right)=z_{2D}\left(n\right)+{\beta 1}_{2D}*ambivalence\left(r\right)\\ +{\beta 1}_{2D}*base\;bias\left(r\right)+{\beta 1}_{2D}*run\left(r\right)+\epsilon_{2D}\left(r\right)\\ \left[model\;2E\right]\\ symp\;state\left(r\right)=z_{2E}\left(n\right)+{\beta 1}_{2E}*ambivalence\left(r\right)\\ +{\beta 1}_{2E}*norm\;rate\left(r\right)+{\beta 1}_{2E}*run\left(r\right)+\epsilon_{2E}\left(r\right)\\ \left[model\;2F\right] \end{array}$$

Where relevant, *t* = *trial, r* = *run, n*=*subject*

In models 1A-B, 2A-B and 2E-F, *symp state* relates to the sympathetic state from both the offer and commit phase of trials, i.e., averaging offer PEP and commit PEP (see *Estimates of sympathetic state - PEP)*, while models 2C and 2D model offer PEP and commit PEP separately.

### Raw Data Depiction

**Figure 2:**
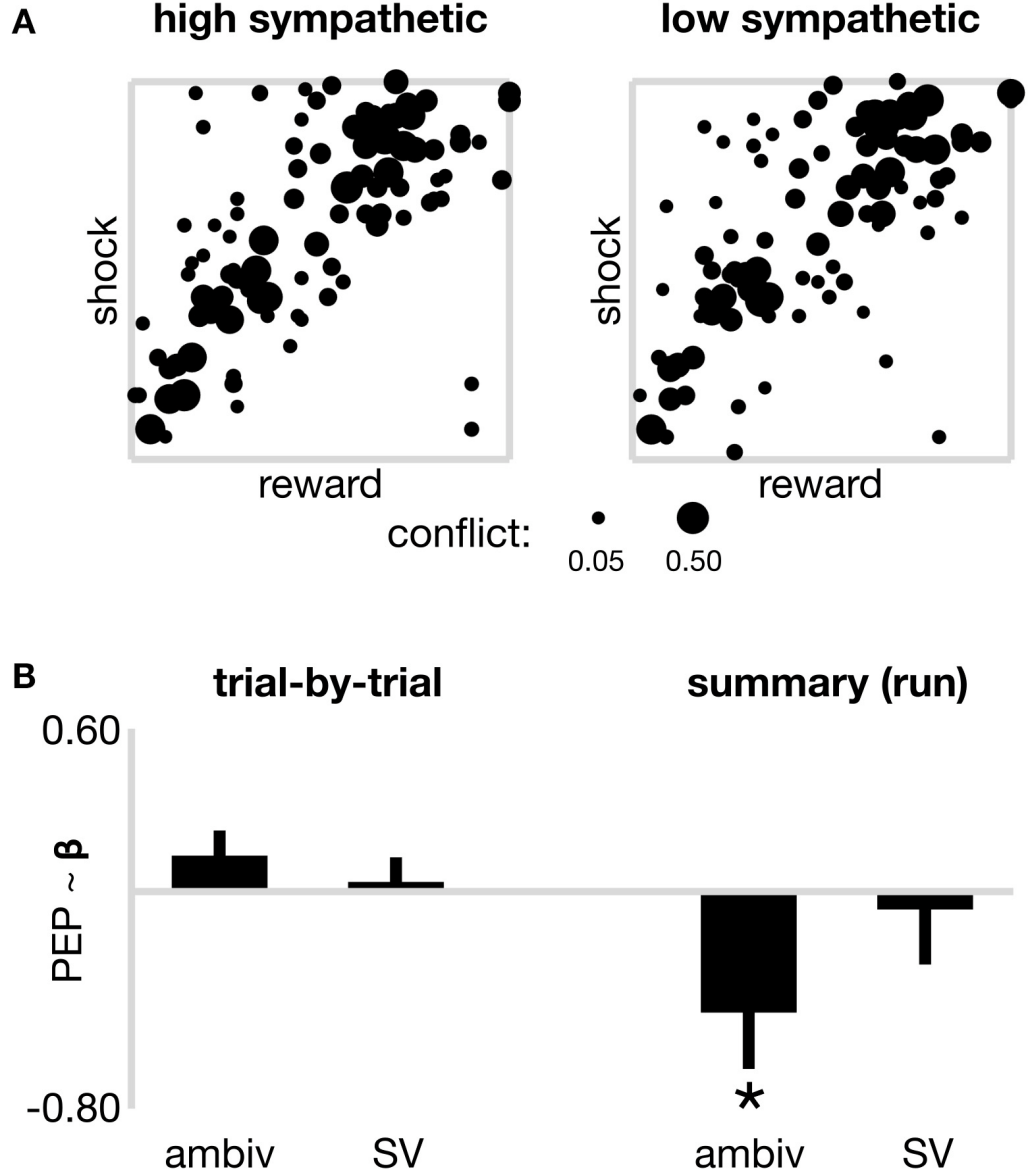
Sympathetic drive and decision parameters. **(A)** Raw data from all participants at each coordinate of decision space, to illustrate the degree of decision ambivalence separately for high and low sympathetic state. Pooled ambivalence was assessed at each coordinate and depicted by the width of the dots, theoretically ranging from 0 (subjects always responded the same way, i.e., approach or avoid) to 0.50 (subjects approached as often as they avoided), however only coordinates that present non-zero ambivalence are included in the plot. **(B)** Models of sympathetic state (PEP) across two time horizons. On the left, trial-by-trial sympathetic state is not significantly associated with the ambivalence or subjective value (*SV*) of individual offers. On the right, sympathetic state (summarized by run) is negatively associated (*p < 0.05) with a summary ambivalence estimate. The nature of this negative correlation is such that increased sympathetic activation (shorter PEP) is present during runs of lower ambivalence (consistent with the more tightly distributed cluster of coordinates displaying non-zero ambivalence in Panel **A**–left). No association observed between summary sympathetic drive and summary subjective value (*SV*). Error bars reflect the standard error of beta estimates from linear mixed effects models fitted with a random intercept.

We graphically depict raw data of the ambivalence arising at each coordinate of decision space in Figure 2A, separately for high and low sympathetic activation. We made this plot by first sorting each subject's set of trials by PEP; trials below median PEP were considered high sympathetic, while those above median PEP were considered low sympathetic. We then merged all participants' high sympathetic trials into one dataset and all low sympathetic trials into another. Then separately for each dataset, we computed ambivalence for each coordinate of decision space, using:

(4) $$\begin{array}{l} ambivalence_{reward,cost}=0.5-|0.5-P\left(N\right)| \end{array}$$

Where *P*(*N*) is the proportion of approached offers at this coordinate, pooling all participants' trials together. This formula yields a value ranging from 0 (low ambivalence; participants always responded in the same manner, approach or avoid) to 0.50 (high ambivalence; participants approached as often as avoided).

### Neuroimaging–Protocol

MRI data were recorded with a 64-channel phased-array head and neck coil using a 3T Magnetom Prisma Fit (Siemens, Germany). Anatomical data were high-resolution 0.94 mm isotropic T1- (TR=2,500 ms, TE=2.2 ms, FA=7, FOV=241,mm) and T2^*^-weighted (TR=3,200 ms, TE=570 ms, FOV=241 mm) sagittal slices of the whole brain. Functional images, recorded during each run of the approach-avoidance task, were T2^*^-weighted (TR=910 ms, TE=32 ms, FA=52, FOV = 192 mm, multiband factor 4) echo planar gradient-echo images, recorded with 58 channels. Each functional image acquired 64 coronal slices, perpendicular to the AC-PC plane (3 mm thick; 3 × 3 mm in-plane resolution). Coronal acquisition reduces artifact when recording simultaneous EKG/ICG (Cieslak et al., 2018).

### Neuroimaging–Pre-Processing

Anatomical images were skull-stripped using the Advanced Neuroimaging Tools (ANTs) software (Avants et al., 2011). Each functional series was trimmed of the first ten images and then skull-stripped (BET) (Smith, 2002), motion-corrected (MCFLIRT) (Jenkinson et al., 2002), spatially smoothed (Gaussian kernel; FWHM=5 mm), de-meaned (grand-mean of the entire 4D data-set, single multiplicative factor) and temporally high-pass filtered (Gaussian-weighted least-squares linear fit, σ =50s), all using FMRIB's Software Library (FSL; Smith et al., 2004). Each functional series was registered to the subject's anatomical image, and then to a standard template (Montreal Neurological Institute (MNI) 2 mm averaged 152; FSL (FLIRT); Jenkinson and Smith, 2001; Jenkinson et al., 2002). Registration was then refined with a non-linear registration (FSL (FNIRT); 10 mm warp resolution; Andersson et al., 2007a,b).

### Neuroimaging–a Priori ROI and Statistical Analysis (GLM)

Given that our behavior-sympathetic-neural hypothesis specifically queries the relation between vmPFC and sympathetic perturbations during value-based ambivalence, we restricted our analyses to task-related activation within this region of interest (ROI). We defined the vmPFC ROI using the Harvard-Oxford cortical structural probabilistic atlas (https://fsl.fmrib.ox.ac.uk/fsl/fslwiki/Atlases) and do not report incidental statistical findings from elsewhere in the brain.

We fitted a modified version of a general linear model (GLM) used in Shapiro and Grafton (2020) to the functional time series of each voxel in the brain. The design matrix contained a row for each functional image and a column for each of nine categorical regressors and eight continuous regressors. All regressors were convolved with a canonical gamma hemodynamic response function. Categorical regressors accounted for neural activation associated with different aspects and phases of trial-by-trial choices. We first determined *SV* for each trial (see *Methods: Estimating subjective value and ambivalence–trial-by-trial)*, then separated each subject's trials into “approach” and “avoid.” We next median split both “approach” and “avoid” trials separately, based on their level of ambivalence (*SV*^2^). Respectively, this allowed the “on” portions of GLM regressors to separately cover (i) the offer phase when approaching low ambivalence trials (ii) the commit phase when approaching low ambivalence trials (iii) the offer phase when approaching high ambivalence trials (iv) the commit phase when approaching high ambivalence trials (v) the offer phase when avoiding low ambivalence trials (vi) the commit phase when avoiding low ambivalence trials (vii) the offer phase when avoiding high ambivalence trials (viii) the commit phase when avoiding high ambivalence trials and (ix) payout phase. These nine regressors serve to remove decision derived modulations from the neural signal (i.e., boosting the signal-to-noise of sympathetic derived modulations). In each case the “offer” and “commit” phases are time-locked to the segments of trials. We will therefore not present or discuss their results, and orient interested readers to Shapiro and Grafton (2020). Our regressors of interest were instead continuous regressors that served to account for neural activation associated with sympathetic activity that mapped onto different aspects and phases of trial-by-trial choices. Respectively, the “on” portions of these regressors again covered (x) the offer phase when approaching low ambivalence trials (xi) the commit phase when approaching low ambivalence trials (xii) the offer phase when approaching high ambivalence trials (xiii) the commit phase when approaching high ambivalence trials (xiv) the offer phase when avoiding low ambivalence trials (xv) the commit phase when avoiding low ambivalence trials (xvi) the offer phase when avoiding high ambivalence trials and (xvii) the commit phase when avoiding high ambivalence trials. “On” portions of these regressors were scaled by the offer PEP (x, xii, xiv, xvi) or commit PEP (xi, xiii, xv, xvii) value for that trial (see trial-by-trial estimates of sympathetic state, above). PEP values were demeaned separately for each regressor before scaling.

We estimated contrasts between different permutations of regressors (x) to (xvii) to explore sympathetic modulation of neural activity mapping onto specific characteristics of *SV* and ambivalence, separately or collapsed across the offer and commit phases of trials. Specifically, these contrasts were: (I) high vs. low *SV* throughout the entire trial (0.25^*^[x-xiii]-0.25^*^[xiv-xvii]); (II) high vs. low *SV* during the offer phase (0.50^*^[x, xii]-0.50^*^[xiv, xvi]); (III) high vs. low *SV* during the commit phase (0.50^*^[xi, xiii]-0.50^*^[xv,x vii]); (IV) low vs. high ambivalence throughout the entire trial (0.25^*^[x, xi, xiv, xv]-0.25^*^[xii, xiii, xvi, xvii]); (V) low vs. high ambivalence during the offer phase (0.50^*^[x, xiv]-0.50^*^[xii, xvi]); (VI) low vs. high ambivalence during the commit phase (0.50^*^[xi, xv]-0.50^*^[xiii, xvii]); (VII) low vs. high ambivalence throughout the entire trial of approached trials (0.50^*^[x, xi]-0.50^*^[xii, xiii]); (VIII) low vs. high ambivalence during the offer phase of approached trials ([x]-[xii]); (IX) low vs. high ambivalence during the commit phase of approached trials ([xi]-[xiii]); (X) low vs. high ambivalence throughout the entire trial of avoided trials (0.50^*^[xiv, xv]-0.50^*^[xvi, xvii]); (XI) low vs. high ambivalence during the offer phase of avoided trials ([xiv]-[xvi]); and (XII) low vs. high ambivalence during the commit phase of approached trials ([xv]-[xvii]).

We fitted the above GLM to each voxel as part of a three-level analysis. Level 1 fitted the GLM to each subject's runs separately and estimated parameters for regressors [i-xvii] and contrasts [I-XII]. Level 2 combined run-level data (fixed effects) to estimate a subject's overall parameters for each regressor and contrast. Finally, Level 3 combined subject-level data (mixed-effects with subject random effect (FSL–FLAME 1) to estimate group-level means for each regressor and contrast.

Finally, for each Level 3 contrast (I-XII) we identified voxels in vmPFC that yielded parameter values beyond a significance threshold that accounts for multiple comparisons using False Discovery Theory (False Discovery Rate; FDR; Genovese et al., 2002). A critical *p*-value was computed from each contrast's raw parameter and parameter variances using the FDR function in FSL. Given that we tested 12 contrasts, we further penalized these critical *p*-values with a Bonferroni adjustment, i.e., division by 12. However, to reduce the risk of type-II error, we report in the results voxels that exceed both the Bonferroni-adjusted and non-Bonferroni-adjusted critical *p*-values (but always FDR corrected).

## Results

Healthy human participants performed six fMRI runs of an approach-avoidance task. Each run contained 31 or 32 trials (189 in total) and lasted ~5 min. In each trial, participants were offered varying levels of money (reward) for varying levels of painful electric shock (cost), during simultaneously recorded neuroimaging and cardiovascular physiology. Combined EKG/ICG assayed the sympathetic state at each individual heartbeat with the pre-ejection period (PEP; see Methods).

### Behavioral Results Summary

Participants showed a tendency to accept more offers than they rejected, accepting a group average 61.7% of offers [95% CI (57.6%, 65.8%) across subjects]. Separately for each subject, trialwise choice probability *p(approach)* was then modeled as a function of an intercept, the magnitude of reward and the magnitude of cost offered on each trial of the task. These parameters were used to compute each participant's baseline bias and normalization rate (see *Estimating subjective value and ambivalence, trial-by-trial and* Figure 1). At the group level, the bias was significantly positive (μ =12.4; 95% CI [7.00, 18.3] across subjects). However, their normalization rate did not significantly depart 1 (μ =0.96; 95% CI [0.86, 1.06] across subjects). Accordingly, at the group level, after controlling for the baseline bias toward acceptance, participants integrated reward and cost evenly (i.e., 1:1). The current subset (*n* = 22) of participants also showed strong agreement with the larger original sample (*n* = 28) on two other core behavioral variables (See Supplementary Materials: *Behavioral alignment between original and current sample* and Supplementary Figure 1).

### Behavior-Sympathetic Results

Trialwise sympathetic state was summarized by taking the average PEP across heartbeats registered during both the offer and commit phase of each trial (mean of offer PEP and commit PEP; see Methods). A trial-by-trial model (*model 1A* in methods) then first tested whether sympathetic state on a given trial was associated with the objective level of offered reward or cost, looking across all trials and all participants. The model contained a random intercept for each subject, and a categorical nuisance regressor to account for trial number and the order of run in the experiment. The model reported no significant association between sympathetic state and reward, cost nor the interaction term between the two (a proxy for “objective ambivalence”; all *p* > 0.400).

Using separate logistic models for each subject's complete set of trials, we then estimated the *SV* of each individual offer, that is, the net yield stemming from the magnitudes of reward and cost, accounting for the two parameters that underscore *SV* tuning: a subject's baseline bias to accept and their normalization rate of reward and cost (see Figure 1C and Methods). Ambivalence, i.e., the proximity of an offer to a subject's approach/avoid threshold, was estimated by squaring *SV* (see Figure 1C and Methods), with higher ambivalence offers being associated with shorter distances to the threshold.

A second trial-by-trial model (*model 1B* in methods) then tested whether sympathetic state on a given trial was associated with either its ambivalence or *SV*, controlling for nuisance regressors of trial number and run order, again looking across all trials from all participants. This model reported neither a significant effect of ambivalence nor *SV* on sympathetic state (both *p* > 0.15; Figure 2B–left panel). Together these first two models suggest perturbations of value and ambivalence, either objective or subjective, are not closely tracked by the sympathetic system on short time scales (seconds). Notably, the nuisance regressor accounting for trial order was not significant in either model (both *p* < 0.303), however the run order was associated with significantly decreasing sympathetic activation in both models (all marginal run effects *p* < 0.015). Therefore, within run, sympathetics showed no time-on-task effects, but across the experiment, time-on-task was associated with a gradual attenuation to sympathetic state.

In additional analyses, included in Supplementary Materials, we likewise see no relation between objective reward or cost, or subjective ambivalence or *SV* on choice-to-choice sympathetic perturbations; that is, parsing trials into two sets (respectively, all trials where participants approached the offer and those where they avoided) and running *models 1A* and *1B* separately on each set.

We next considered whether sympathetic state (high or low) assayed over a longer time period than individual trials might be related to choice-related model parameters including value-driven ambivalence and *SV*. For each subject, and for each run, we computed a statistic that estimated a “summary” sympathetic state for that run (each run reflecting ~5 min of decision-making on up to 32 trials), in addition to summary variables for objective reward and cost, and the ambivalence and *SV* presented by the run's composite trials. Note that whereas in the first set of models trial-wise *SV* and ambivalence were computed from individualized models that took into account all trials from the participant's experimental session, here separate models were fitted to each run (six models per participant) and the resulting trial-wise *SV* and confidence from within each run were summarized as median scores. We first used a linear mixed effects model (*model 2A* in METHODS) to explore the relationship between summarized sympathetic state, and summarized objective reward and cost. The model contained a random intercept for each subject, and a categorical nuisance regressor to account for the order of run in the experiment. The model reported no significant association between sympathetic state and reward, cost nor the interaction term between the two (a proxy for “objective ambivalence”; all *p* > 0.506). We next ran a model to test whether summarized sympathetic state was associated with summarized ambivalence or *SV* (*model 2B* in METHODS). As depicted on the right panel of Figure 2B, the model reported a significant negative association between sympathetic state and ambivalence (β = −0.450; *s*.*e*. = 0.208; *p* = 0.034). Note that in our analyses both ambivalence and sympathetic state scale negatively, i.e., higher numerical values, respectively, reflect reduced ambivalence (farther distance from the approach/avoid threshold) and reduced sympathetic activation (longer PEP). This negative association therefore shows that increased sympathetic activation aligns with decreased ambivalence, and vice versa, on time scales traversing several minutes. This finding therefore suggests that decision making aggregated over many trials shows more consistent value-action mapping during periods of sustained elevated sympathetic state, consistent with the summarized raw data in Figure 2A, which depicts a tighter spread of coordinates displaying non-zero ambivalence during high sympathetic activation (left panel) vs. low sympathetic (right). In a control ANOVA (Supplementary Materials) we do not see ambivalence vary systematically across runs. We further did not observe a significant association between sympathetic state and *SV* (β = −0.066; *s*.*e*. = 0.203; *p* = 0.745) and the nuisance regressor again suggested marginal effects of time-on-task, for runs 5 and 6 (both *p* < 0.01), but only in the subjective value *model 2B* (objective *model 2A* all *p* > 0.161).

Previous studies of sympathetic changes during choice tasks have suggested that they are associated with allostatic related mobilization (see Richter et al., 2016 for review). These findings motivated us to test whether the observed sympathetic-ambivalence associations in our summary model were due to sympathetic perturbations occurring within a specific time interval of each trial. We re-ran our summary model, separately for PEP values averaged across only heartbeats during the offer phase of trials (*model 2C*–offer sympathetics model), and again for values averaged across only heartbeats during the commit and feedback phase of trials (*model 2D*–commit sympathetics model). Mobilization-based accounts would predict that the sympathetic changes are related to the commit phase of the trial as this is the time period when one must initiate an action to enact their decision. Consistent with this, we found that the sympathetics had a significant (negative) association with ambivalence only during the commit phase (β = −0.410; *s*.*e*. = 0.201; *p* = 0.044), but no significant association with ambivalence during the offer phase (β = −0.352; *s*.*e*. = 0.206; *p* = 0.091). This suggests the association between the sympathetic system and decision ambivalence might be more relevant during action execution than during choice deliberation.

Finally, we also considered whether sympathetic state, summarized over each run, was associated with two other measures of choice behavior besides composite *SV*. First, we tested if each participant's sympathetic drive for a given run could be described by a baseline bias, i.e., if a baseline level of reward were needed for any magnitude of cost to be approached, or conversely if a baseline level of cost would be tolerated regardless of the offered reward. We first ran a model that kept the ambivalence regressor, but replaced *SV* with a regressor that measured constant bias (*model 2E)*. This model again showed a significant negative association between ambivalence and sympathetic state (β = −0.483; *s*.*e*. = 0.212; *p* = 0.025), but no significant sympathetic association for the bias parameter (β = 0.214; *s*.*e*. = 0.249; *p* = 0.392). Second, we tested whether sympathetic state was associated with the normalization rate (*model 2F)*, i.e., if subjective value of reward units were valued higher than that of cost units, or vice versa. Replacing *SV* with normalization rate in the summary model, we again observed a significant negative association between ambivalence and sympathetic state (β = −0.614; *s*.*e*. = 0.228; *p* = 0.008), and a marginally significant negative association between sympathetic state and normalization rate (β = −0.415; *s*.*e*. = 0.239; *p* = 0.085). The nature of this negative association would align an increased sympathetic state with a greater weighting of reward value relative to cost.

Note that our significant association between summary sympathetic state and ambivalence would not survive correction for the multiple statistical tests performed (*p*-values ranging from 0.025 to 0.044, multiple linear mixed effects models). To assist future studies assessing the robustness of this finding we have computed a Bayesian estimate of the association's parameter (posterior μ = −0.340; posterior σ =0.203; see Supplementary Materials: *Bayesian parameter of ambivalence-sympathetic association*). These posterior parameters can configure a Gaussian prior for update with a new dataset.

In summary, our extensive analysis of potential behavior-sympathetic interactions implicate a role for sustained sympathetic activity with consistent value-action mapping. When sympathetic state is elevated over a sustained period, it is more likely that a person will be committing to approach offers of positive subjective value and avoid offers of negative subjective value. In contrast, sympathetic activity appears to be minimally associated with subjective appraisals of an offer's value. These results point to the sympathetic system providing allostatic support in value-based ambivalence, most likely operating on decision enactment during the commit phase.

### Behavior-Sympathetic-Neural Results

vmPFC is functionally linked to both decision ambivalence and allostasis, and appears to play a role in regulating fear responses to appropriate stimuli. We therefore hypothesized that in addition to its modulation by decision parameters, vmPFC might show activations consistent with it regulating the sympathetic response. Our first set of results, proposing an adaptive relation between sympathetic state and ambivalence, refines this prediction to vmPFC activations attenuating sympathetic state during low ambivalence. To test this association, we modeled the neural activity of each voxel in the brain using a general linear model (GLM) that categorically accounted for variance in neural responses ascribed to the exhaustive combination of the choice (approach or avoid), ambivalence (high or lo) and phase (offer or commit) of each trial, yielding eight regressors. We then added a complementary set of eight regressors, parametrically weighted by sympathetic drive corresponding to the onsets and durations of trials for each of the eight categorical decision parameters.

**Figure 3:**
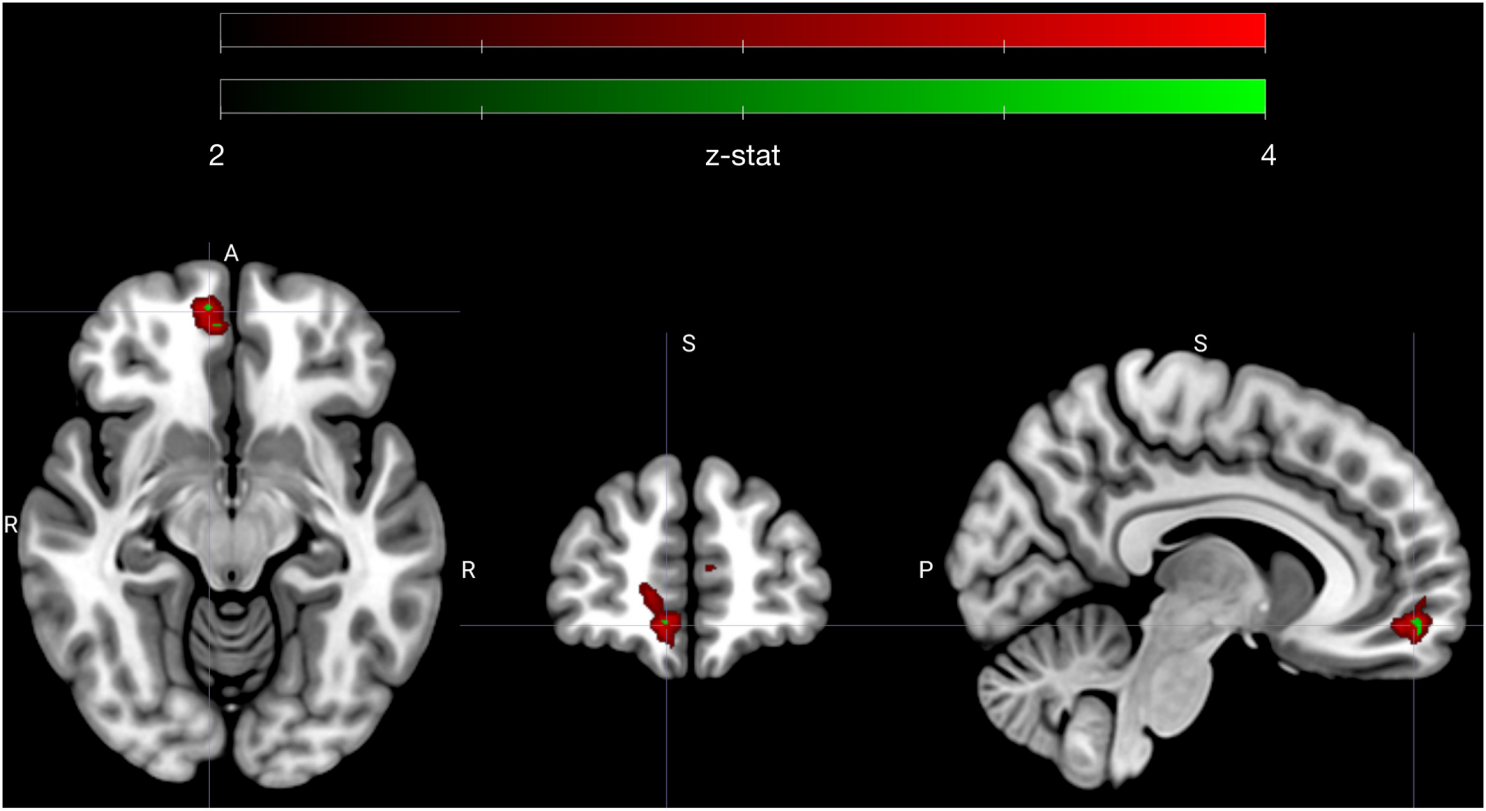
Early avoidance-specific sympathetic parsimony in vmPFC. Figure highlights a cluster of voxels in right vmPFC that encode attenuation of sympathetic state preferentially for low ambivalence and specifically during the offer phase of avoided trials (see contrast XI in methods). Voxels depicted in red (*n* = 175) met an adjusted significance threshold using FDR correction in the vmPFC region (red map) and voxels in green (*n* = 8) further met a significance set by a Bonferroni correction to account for all tested contrasts (*n* = 12). Cluster is centered on MNI coordinate [7.3 54.3 −11.3], the centroid of Bonferroni significant voxels. Colormap ranges from z-statistic of 2 to 4.

The sympathetic weighted parametric regressors were used to make contrasts that probed whether voxels that covaried with our sympathetic assay (PEP) were more sensitive to either high vs. low subjective value, or low vs. high ambivalence. To test if any correlations between sympathetics and neural activity were specific to approach or avoid behavior, we tested all contrasts with only the approach trials, only the avoid trials and for all trials. As with our behavioral models, we also tested whether the contrasts applied to specific moments in trials (i.e., the offer or commit period), or showed no temporal specificity. We limited all statistical comparisons to a vmPFC ROI, which was defined a priori. Out of 12 resulting contrasts, one yielded a significant cluster of voxels (*n* = 175) in vmPFC that passed a significance threshold that accounted for multiple comparisons using false discovery rate (FDR) correction in vmPFC. A subset of these voxels (*n* = 8) were also significant at a level that adjusted FDR thresholds with Bonferroni correction to account for the 12 tested contrasts. The specific contrast (see contrast XI in methods) compared the offer period of low vs. high ambivalence trials that were avoided. Highlighted in Figure 3, the cluster is clearly centered in the right ventromedial frontal cortex. The mean (standard deviation) z-statistics were 3.14 (0.23) for the FDR correction cluster, and 3.67 (0.06) for the Bonferroni sub-cluster. The positive z-statistics indicate that this region of vmPFC is more actively coupled with the sympathetic response during low ambivalence trials than during high ambivalence trials. Further, the sign of the relationship is such that greater activity in vmPFC is associated with a more attenuated sympathetic state.

In other words, vmPFC activates early in avoided low ambivalence trials, in line with the degree of attenuation of sympathetic state. All other contrasts failed to reach the threshold set by the FDR adjustment in vmPFC, including comparisons of high vs. low subjective value at each phase, and the analogous contrast between high and low ambivalence during the offer phase of approach trials. We therefore specifically see sympathetic-neural associations related to low ambivalence avoidance and not a more global attenuation of sympathetic stress during negative *SV* or during low ambivalence of either valence.

The above GLM estimates trial-by-trial sympathetic-neural relations across three dimensions: modulations separated by the selected action (approach-avoid), for different levels of ambivalence (high-low) and at different periods of time in the offer (early-late). We selected this GLM to align our exploration of sympathetic-neural relations with the behavior-neural design of Shapiro and Grafton (2020). Note, however, that the primary finding from the behavior-sympathetic analyses (above) was on a different time horizon, i.e., summary values of sympathetic state associated with longer periods of consistent decision making. We therefore conducted a summary run-by-run analysis, which tested if differences in vmPFC activity between low and high ambivalence scaled according to the summary sympathetic state of each run. However, this analysis (*single-run average contrast with additional covariate*; included as a Supplementary Material) did not return any significant voxels in vmPFC.

Thus, in summary, our behavior-sympathetic-neural results reveal a potential role of vmPFC, which operates on a short time horizon by modulating sympathetic state at a specific phase of individual trials. Early in trials, as subjects make appraisals of offers that they will eventually avoid with low levels of ambivalence, vmPFC activity is associated with an attenuated sympathetic state. This finding is consistent with an action-specific (avoid) resource parsimony role for vmPFC in value-driven ambivalence allostasis.

### Supplementary Analyses

We include two additional exploratory analyses in Supplementary Materials that were requested by reviewers and not guided by our primary hypothesis.

First, for illustrative purposes, we report activations of our twelve contrasts in different ROIs that include more regions of the medial frontal lobes (specifically anterior cingulate cortex, paracingulate gyrus and subgenual cortex) and also the insula. We report the mean parameter estimates in all voxels of each region, in addition to the mean variance of parameter estimates. (See Contrast estimates in other ROIs and Supplementary Table 1).

Second, we tested whether the intensity of our sympathetically modulated vmPFC activity covaried across subjects with intensities in other brain regions important for value-based decision making (dorsolateral prefrontal cortex, anterior cingulate cortex and right amygdala). Results suggest sympathetically modulated vmPFC activity might be related to activity in these regions, across a range of choice actions (accept/reject) and time frames (offer period/commit period). (See *Between subject associations of activation associations* and Supplementary Figure 2).

## Discussion

As organisms approach or avoid cost-bearing reward, value-driven ambivalence scales with the net yield's proximity to subjectively tuned decision thresholds. Consistent value-action mapping forms a cleaner discretization of an organism's decision space created by more approaches to marginally positive offers and more avoidance of marginally negative offers. In ever-changing environments, where low-ambivalence decisions may be rare or transiently available, it accordingly might behoove an organism to develop an efficient neural network that only mobilizes physiological resources to resolve value-driven ambivalence where needed, and optimize behavior over a sustained period of goal-directed choices. Crucial for this network's efficiency would be the organism preserving control of physiological mobilization, to attenuate needless energy expenditure in situations of more immediately identifiable value-action policy. Combining cardiac-autonomic and neural recordings during an approach-avoidance task, we provide the first data that supports both branches of this allostatic model of value-driven ambivalence in humans.

### Sympathetic Mediated Allostasis During Value-Driven Ambivalence

In a first set of analyses we identify behavior-sympathetic relations underscoring value-driven ambivalence, and reveal a unique association between mobilized sympathetic state and consistent value-action mapping. This association was not observed on a trial-by-trial basis. Rather, an alignment emerged when both sympathetic state and decision ambivalence were measured on aggregate, over several minutes of decision making. In the context of our task, sympathetic drive therefore aligns with an adaptive response. We further did not observe any associations between sympathetic drive and high ambivalence, either across short (moment-to-moment fluctuations) or longer (summarized) time horizons. Sympathetic state therefore did not track a homeostatic response to the phenomenology of difficult decisions. Sympathetic modulation was further unique to ambivalence, and not the objective availability of reward or cost, nor ensemble subjective value. Also, while our findings uncovered no sympathetic association with a specific action-in-ambivalence, we did observe evidence that the ambivalence-linked sympathetic contributions might dominate during offer commitment rather than during offer appraisal.

Temporally-specific mapping of sympathetics onto action commitment frames our result within a model proposed by Wright (1996), that integrates both active coping (Obrist, 1981), and motivational intensity theory (Brehm and Self, 1989), and predicts that sympathetic mediated effort mobilization should scale positively with parameters such as task difficulty and reward importance during goal-directed behavior (Richter and Gendolla, 2009). Later studies (reviewed in Richter et al., 2016) have supported this model using a number of cognitive and motor tasks. We extend this literature by using an approach-avoidance task, which allows both reward and cost to vary, moment-to-moment. Importantly, the task design allowed us to decouple value from ambivalence. A classic mobilization finding is that subjects performing a cognitive task, aware that their performance might earn a bonus, but not aware of the exact required standard, will show greater sympathetic drive when the promised bonus is of more value (Richter and Gendolla, 2009). While this suggests sympathetic state scales with reward availability, drive may also (or instead) scale with task settings positioned farther from a decision-to-mobilize threshold; one that traverses a two-dimensional reward-effort decision space. The approach-avoidance paradigm, and its ability to gauge ambivalence arising from reward integrating with qualitatively different cost dimensions, including cognitive (task difficulty), nociceptive (pain), physiological (vigor) and economic (monetary loss), is a promising means toward parsing the separate influences of value and ambivalence on effort mobilization.

Our findings are also consistent with recent behavior-sympathetic data we reported on human subjects making approach-avoidance choices in a time-constrained sequential foraging paradigm (Dundon et al., 2020). In this prey-selection experiment, we likewise did not see sympathetic state track either objective or subjective levels of reward or cost on individual trials. Instead, sympathetics were implicated in an adaptive response that aligned with subjects making the appropriate change to their behavioral policy during a downard change in a patch's profitability (i.e., as the reward rate lowers, it becomes optimal to capture more high-cost prey). Both these data and our current findings can be unified by proposing that sympathetics facilitate an adaptive response in the face of different abstractions of ambivalence. In the present experiment, ambivalence arises from an offer's proximity to a decision boundary that was characterized by temporally invariant reward (money) and cost (pain). In the prey-selection task, ambivalence arises from capture opportunity costs becoming more ambiguous during a gradual change in estimated environmental dynamics. Together, both studies' findings suggest sympathetic drive is not an urgency signal to act indiscriminately, but that mobilization may also be in some way linked to more resolute traces of value or state estimations.

### vmPFC as a Control Node in Ambivalence Allostasis

In our behavior-sympathetic-neural analyses we identify a potential control node for sympathetic mobilization in vmPFC. Operating on a short time horizon, voxels in this region were modulated by sympathetic state at a specific phase of individual trials. As participants appraised offers they would eventually avoid, vmPFC countercyclically tracked sympathetic state selectively on trials presenting low ambivalence offers. In other words, when participants were sizing up lousy offers they would surely avoid, activation in vmPFC was increasing as sympathetic state was attenuating. vmPFC did not track sympathetics in any other contrasts that exhaustively tested pairwise permutations of selected action (approach-avoid), levels of ambivalence (hi-low) and phase in the offer (early-late). Nor did we observe vmPFC involvement in a run-by-run analysis analogous to the behavior-sympathetic finding.

The temporal specificity of vmPFC's sympathetic involvement aligns our findings with animal models that have identified this region as a key node within a cortical network that mediates rest-activity states in an integrated medullary-midbrain-spinal circuit, in preparation for situations that might require an alert or effortful action (Schulkin and Sterling, 2019). Prior to action, through its projections to both motor areas and central pattern generators for autonomic activity (Dum et al., 2016; 2019), vmPFC can both indirectly (via motor projections) and directly modulate sympathetic state. However, the action specificity of our finding further suggests that vmPFC's sympathetic involvement may not always be behaviorally symmetric. This is consistent with frameworks that consider approach-avoidance behavior under an ethological framework whereby approaching and avoiding could be underscored by disparate networks that evolved separately to optimize qualitatively different state-action transitions (Hayden, 2018). For example, when an organism forages for food or prey, its default state is an outwardly oriented exploration. Approach behavior actively departures from the status quo, orienting neural and physiological states toward capture or consumption, while avoidance behavior passively maintains the exploratory state (Hayden, 2018). Indeed, vmPFC has shown action-contingent qualitative differences in neural coding during foraging, i.e., coding value when approaching, but environmental richness when avoiding (Kolling et al., 2012).

### vmPFC-Amygdala Connectivity and Learning Deficits in Anxiety

Another route for vmPFC to modulate sympathetic state might exist via its projections to the amygdala. In particular, the basolateral amygdala is reciprocally connected to vmPFC, with evidence coming from both animal lesion studies (Carmichael and Price, 1995; Ghashghaei and Barbas, 2002; Ghashghaei et al., 2007) and human structural connectivity data (Croxson et al., 2005; Johansen-Berg et al., 2008; Bracht et al., 2009). The central nucleus of the amygdala has fewer direct synapses with vmPFC, and instead appears to play a key role in regulating autonomic behavior via projections to hypothalamic and brainstem structures (Bohus et al., 1996). This provides a potential indirect limbic pathway between vmPFC and autonomic control, i.e., vmPFC to basolateral amygdala to central nucleus.

This network is also relevant to anxiety, given that vmPFC-amygdala connections have been studied in the context of both anxiety behavior itself (Felix-Ortiz et al., 2016) in addition to extinction learning (Falls et al., 1992; Lu et al., 2001; Milad and Quirk, 2002). Extinction is the process by which an organism learns that a conditioned stimulus is no longer paired with a previous aversive outcome, and is considered a complementary anxiety phenotype to fear generalization (Dunsmoor and Paz, 2015). In rodent models vmPFC activity scales negatively with pervasive maintenance of aversive beliefs (Milad and Quirk, 2002), mirroring its dysfunctional role with human anxiety participants in fear generalization, i.e., reduced activation during “safe” stimuli.

### A Unifying Role for Sympathetics: State Consultation

Our primary finding is that sympathetic drive aligns with consistent value-action mapping, and that vmPFC activity is linked with attenuating sympathetic state early in low ambivalence avoidance. We do not conclusively establish how vmPFC might modulate sympathetic state, or whether it does so via more than one pathway. However, one possibility is that vmPFC's sympathetic modulation involves a pathway to the autonomic system via the amygdala, and thus involves neural architecture that underscores anxiety behaviors and a similar learning mechanism to fear generalization (extinction learning). Sympathetics might be linked with the cognitive mechanisms of our approach-avoidance task and, further, with the mechanisms of both fear generalization and extinction learning by its modulation of a parameter of reinforcement learning: state representations. State representations are present when an organism forms an abstract representation of a task's structure. For example, fear generalization is ultimately a failure to accurately form a state representation, i.e., that only one of two stimuli will ever lead to an aversive outcome. Tasks may further involve more than one state. Therefore, learning and identifying states allows an organism to quickly recruit the appropriate action policy (if it gets cloudy bring an umbrella–don't wait for it to rain). Approach-avoidance tasks might be conceptualized as a fairly simple two-state task, in which subjective value is either above zero (policy: approach) or below zero (policy: avoid). However, ambivalence adds an increasingly gray area between these two states, where policy is less clear. Since state representations themselves are more likely coded in the orbitofrontal cortex (Wilson et al., 2014), vmPFC's role is likely to be more of a gain controller than a cache, regulating the sympathetics to mobilize in an effort to more resolutely consult state representations (state consultation) and arrive at more definitive policy during action selection. Emerging computational evidence suggests that state representations may indeed be impaired in anxiety, as evidenced by a stronger tendency to engage in lose-switch (i.e., probabilistic) behavior (Huang et al., 2017). Together, this model would therefore predict fear-generalization in anxiety participants would be accompanied by dysfunctional sympathetic drive during “safe” stimuli, and the degree of sympathetic dysregulation may scale with vmPFC activity, while possibly preserving function in orbitofrontal cortex.

### Limitations and Future Recommendations

The primary limitation of our study is that all findings are correlational and we thus cannot make causal claims about sympathetic drive and behavior, or the vmPFC and sympathetic drive. In addition, we found no associations between vmPFC and any increases to sympathetic state, either during single trials or in an analogous run-by-run model. It could be that vmPFC's association with sympathetic drive is more tightly coupled with its role in the actual decision process, leaving no residual variance for sympathetic regressors in the GLM. If this is the case, future studies might discover ways to separate neural and physiological responses more definitively. Alternatively, drive may lead to modulations in brain regions outside vmPFC, such as motor cortex (which also synapses with adrenal medulla; Dum et al., 2016) or sites along the insula, an important node in allostasis (Kleckner et al., 2017). Since our hypothesis was restricted to exploring vmPFC function, probing these other sites will require a priori hypotheses and a new data set. Also, low probability payout trials were preferred to administering painful electric shock while subjects were in an fMRI environment wearing an array of EKG and ICG electrodes. However such payment structure may have created a confound when interpreting our behavior-sympathetic-neural results in our task. Since reward temporally discounts to a higher degree than cost (Murphy et al., 2001), our task possibly created a context which increased the subjective weighting of cost during individual offer appraisal. While this is unlikely to have affected our behavior-sympathetic findings, it offers an alternative value-based explanation to vmPFC's specific role in low ambivalence avoidance, i.e., vmPFC may be modulated by sympathetics in line with subjective value but, due to overweighting of cost, values did not reach a threshold positive level to modulate sympathetic state. Finally, our task only explored one dimension of reward and cost, and further imposed no identifiable environmental dynamics. Our task was in some respects similar to an abstract economic choice task, i.e., imparting a delayed passive cost (shock) in order to obtain a delayed monetary reward. However, the strong relation between sympathetic drive and physical effort and mobilization likely interacts with the broader evolution of approach-avoidance networks that originally mediated choices that imparted immediate physical costs (active) in order to obtain immediate food reward. As Hayden (2018) comments: decision circuitry did not evolve ‘in bloodless terms’. Further investigations can therefore be oriented toward a number of combinations across a multi-dimensional research space described by the passivity (active/passive) and immediacy (immediate/delayed) of rewards and costs. Sympathetic modulations might be a global phenomenon for this entire space, or refined to decisions with a greater emphasis on the active/immediate contexts in which the networks evolved. Such studies could test if neural-sympathetic associations accordingly map onto different brain regions, depending on the composite dimensions of decision space, and the degree of underlying learning or survival requirements.

## Data Availability Statement

The raw data supporting the conclusions of this article will be made available by the authors, without undue reservation.

## Ethics Statement

The studies involving human participants were reviewed and approved by Institutional Review Board at The University of California, Santa Barbara. The patients/participants provided their written informed consent to participate in this study.

## Author Contributions

AS and SG designed the study. AS, VB, and GO oversaw data collection and pre-processing. ND analyzed data and wrote the draft. ND, AS, VB, GO, and SG revised the draft. All authors contributed to the article and approved the submitted version.

## Conflict of Interest

The authors declare that the research was conducted in the absence of any commercial or financial relationships that could be construed as a potential conflict of interest.
