# Supplementary material for: Ventromedial Prefrontal Cortex Activity and Sympathetic Allostasis During Value-Based Ambivalence
Source: Front Behav Neurosci. 2021 Feb 22;15:615796. doi: 10.3389/fnbeh.2021.615796 (PMC7937876; doi:10.3389/fnbeh.2021.615796)
Supplement: Supplementary file 1 [file Table_1.DOCX]

*Supplementary Table 1: Contrast estimates in other ROIs*

| contrast | vmPFC | ACC | PG | SC | rIns | lIns |
| --- | --- | --- | --- | --- | --- | --- |
|  |  |  |  |  |  |  |
| I | 0.228 (0.617) | 0.000 (0.410) | 0.147 (0.618) | 0.071 (0.224) | 0.024 (0.377) | -0.079 (0.344) |
| II | -0.349 (0.860) | -0.276 (0.634) | -0.266 (0.891) | -0.575 (0.474) | -0.406 (0.649) | -0.352 (0.623) |
| III | 0.712 (2.90) | 0.101 (2.17) | 0.381 (3.19) | 0.647 (1.29) | 0.329 (2.00) | 0.090 (1.76) |
| IV | 0.683 (0.738) | 0.634 (0.463) | 0.706 (0.663) | 0.371 (0.268) | 0.500 (0.484) | 0.606 (0.340) |
| V | 0.730 (0.840) | 0.887 (0.742) | 1.03 (0.956) | 0.054 (0.447) | 0.493 (0.770) | 0.577 (0.632) |
| VI | 0.632 (2.43) | 0.630 (1.87) | 0.620 (2.54) | 0.801 (1.20) | 0.706 (2.02) | 0.768 (1.54) |
| VII | 0.051 (0.691) | 0.063 (0.463) | 0.151 (0.703) | 0.017 (0.261) | 0.068 (0.411) | 0.087 (0.377) |
| VIII | 0.354 (1.27) | 0.796 (0.935) | 0.598 (1.32) | 0.162 (0.560) | 0.662 (0.838) | 0.788 (0.741) |
| IX | -0.197 (2.80) | -0.635 (2.02) | -0.206 (2.86) | -0.058 (1.31) | -0.476 (1.92) | -0.644 (1.74) |
| X | 2.07 (1.83) | 1.41 (1.04) | 1.58 (1.54) | 1.04 (0.544) | 1.14 (1.04) | 1.31 (0.714) |
| XI | 2.23 (2.40) | 1.82 (1.78) | 2.47 (2.51) | 0.519 (0.994) | 1.10 (1.69) | 1.06 (1.52) |
| XII | 1.45 (5.24) | 1.19 (4.32) | 0.775 (5.71) | 1.64 (2.61) | 1.37 (4.42) | 1.66 (3.52) |
|  |  |  |  |  |  |  |

*Mean activations of contrasts of parameters (fsl - cope) for twelve contrasts tested in primary GLM (see Neuroimaging - a priori ROI and statistical analysis (GLM) in Methods) for regions not included in primary hypothesis. Values in brackets are mean expected variances of contrasts (fsl - varcope). Regions were defined by the Harvard-Oxford cortical structural probabilistic atlas (https://fsl.fmrib.ox.ac.uk/fsl/fslwiki/Atlases): vmPFC (frontal medial cortex), ACC (cingulate gyrus - anterior division), PG (paracingulate gyrus), SC (subcallosal cortex), rIns (right insula) and lIns (left insula).*
