# Supplementary material for: Ventromedial Prefrontal Cortex Activity and Sympathetic Allostasis During Value-Based Ambivalence
Source: Front Behav Neurosci. 2021 Feb 22;15:615796. doi: 10.3389/fnbeh.2021.615796 (PMC7937876; doi:10.3389/fnbeh.2021.615796)
Supplement: Supplementary file 2 [file Data_Sheet_1.pdf]

Supplementary materials

GLM with single-run average contrast with additional covariate

In the main manuscript we report vmPFC representing the relationship between sympathetic state and ambivalence, using a GLM that considered dynamics across individual trials. However, our behaviour-sympathetic results suggest only summary sympathetic state aligns with consistent value-action mapping. We therefore conducted an additional analysis to test whether voxels in vmPFC also represented the relationship between sympathetic state and ambivalence over a longer time horizon. We ran a second GLM which preserved the original nine categorical regressors, i.e. (i) the offer phase when approaching low ambivalence trials (ii) the commit phase when approaching low ambivalence trials (iii) the offer phase when approaching high ambivalence trials (iv) the commit phase when approaching high ambivalence trials (v) the offer phase when avoiding low ambivalence trials (vi) the commit phase when avoiding low ambivalence trials (vii) the offer phase when avoiding high ambivalence trials (viii) the commit phase when avoiding high ambivalence trials and (ix) payout phase. We conducted a Level 1 analysis for each individual run of each subject, that also tested three contrasts: (I) low vs high ambivalence throughout the entire trial (0.25*[i,ii,v,vi]-0.25*[iii,iv,vii,viii]); (II) low vs high ambivalence during the offer phase (0.50*[i,v]-0.50*[iii,vii]); (III) low vs high ambivalence during the commit phase (0.50*[ii,vi]-0.50*[iv,viii]). At the Level II level of the analysis (across runs, per subject) we took the six parameters for each of these three contrasts per voxel (i.e., one parameter per voxel for each run) and regressed (separately per subject) them with the summary sympathetic state for its run (median PEP). This yielded three Level 2 parameters that described how each voxel represented the relationship between summary sympathetic state, and low vs high neural ambivalence, for the entire trial (I) or only offer (II) or commit (III) phases. At the Level 3 level we used mixed effects to collapse these Level 2 parameters across subjects but found no voxels in vmPFC that reached significance beyond a level set by FDR correction.

Choice-by-choice behaviour-sympathetic models

We conducted additional behaviour-sympathetic analyses to probe whether sympathetic drive mapped onto specific actions on a moment-to-moment basis. We created two datasets, which only contained either trials that participants approached, or trials that they avoided. We first fitted the same design matrices from the trial-by-trial *model 1A* in the main paper which tested whether sympathetic state on a given action was associated with the objective level of offered reward or cost, looking across all trials and all participants. Neither the model on approach trials (all p-values>0.277) nor on avoid trials (all p-values>0.214) returned any significant parameters. We next fitted the same design matrices from the trial-by-trial *model 1B* in the main paper which tested whether sympathetic state on a given action was associated with either the ambivalence or level of subjective value on a given trial, looking across all trials and all participants. Neither the model on approach trials (all p-values>0.281) nor on avoid trials (all p-values>0.178) returned any significant parameters.

Control ANOVA for summary ambivalence

In order to make sure that the associations observed in the behaviour-sympathetic models - that summary sympathetic state aligned with a summary of ambivalence - were not confounded by a chance occurrence of systematic fluctuations in value-mapping consistency across runs, we conducted a one-way repeated measures ANOVA, with a factor of run (1-6) and dependent variable of summary ambivalence, i.e., the median distance of each run's offer to the decision boundary computed for that run. This ANOVA did not return a significant result ($F=0.86, df=5,105, p<0.511$).

Control gLME for effects of payout awareness

One feature of the paradigm is that the payout screen shows the accumulated reward and cost at that moment. It's possible that this information would impact the following trial, for e.g., participants might become more cautious if they have just been reminded that they have a large amount of punishment already accumulated. We therefore tested the impact of cumulative accepted reward and shock on the trial immediately following a payout trial. We modeled choice (p(accept)) on trial t+1 (where payout was given on trial t) as a function of an intercept (a) and four regressors that accounted for:

B1: total reward accepted so far (i.e., up to and including trial t)
B2: total shock accepted so far (i.e., up to and including trial t)
B3: reward on the current trial (i.e., trial t+1)
B4: shock on the current trial (i.e., trial t+1)

Given the small number of payout trials per subject (approx 10) we used a generalized linear mixed effects (gLME) model on a pooled dataset of all participants. The model estimates a single group estimate for each parameter (B1-B4), and an individual estimate for the intercept (a) per subject (random intercept model). We fitted the model using the fitglme function in MATLAB, specifying a Poisson distribution, log link function and Laplacian approximation.

The model showed a significant positive effect for B3 ($B=0.015, s.e.=0.003, p<.001$), and significant negative effect for B4 ($B=-0.016, s.e.=0.003, p<.001$), with no significant effects for B1 or B2 (both p-values > .515). Choice on the trial immediately following a payout was therefore solely modulated by the immediate offer, and not the reward or punishment accumulated to that point, or "in the bank".

Behavioural alignment between original and current sample

Our current sample (*n*=22) comprises the entire subset of participants from a previous sample (Shapiro & Grafton, 2020;n=28) who performed the approach-avoidance task under a simultaneous fMRI/EKG/ICG protocol (remaining six participants were fMRI only). We

established that behavioral data from this subset were similar to the larger group by replicating the two core behavioural analyses of Shapiro & Grafton (2020).

We first compared the range of regression coefficients from each individual's separately fitted logistic choice models (see: *Estimating subjective value and ambivalence, trial-by-trial*, in Methods), i.e., to see that the integration of reward (money) and cost (shock) into choices was comparable between the two groups. Summarized in panel A of figure S1, coefficients from the current sample had group means of 0.208 (reward) and -0.179 (cost), with respective ranges: [0.065,0.547] and [-0.548,-0.051]. Coefficients from the original sample had group means of 0.199 (reward) and -0.170 (cost), with respective ranges: [0.064,0.547] and [-0.547,-0.050]. Reward and cost integration into choices was therefore practically identical between the current and original sample. (Note that error bars in figure S1A reflect the standard error of the mean.)

We next verified that choice reaction times showed a similar inverse quadratic function with respect to value. Such a relationship indicates that subjects were likely experiencing ambivalence for choices of marginal value (i.e., when subjective value (SV) is close to 0), as opposed to easy approaches (positive SV) or easy avoids (negative SV). We used the same procedure as Shaprio & Grafton (2020): we first estimated the quadratic extension of SV of each trial using:

$$quadratic\ SV = [p(Accept) - p(Reject)]^2$$

where p(Accept) and p(Reject) were estimated from the regression coefficients from the separately fitted logistic choice models. We then sorted each subject's trials into 5 equally spaced bins between quadratic SV values 0 (maximum ambivalence) to 1 (always accept or always reject), and modeled mean RTs for each bin across subjects with a linear mixed effects regression (random intercept model). Trials exceeding three standard deviations of the total or bin-wise means were excluded. Quadratic SV had a significantly negative effect on RT ($t=-5.884, p<.001$), indicating that subjects were indeed slower to make choices as quadratic SV approached 0 (maximum ambivalence) and were faster to make choices that presented less ambivalence. This effect was similar to that observed with the original larger sample: ($t=-6.90, p<.001$; see panel B of figure S1 when RT bins are plotted as a function of linear subjective value, for both samples). Note that in addition to establishing the presence of ambivalence during decisions, this inverse quadratic function also verifies that decisions were not wholly formed during the offer period of trials.

Between subject associations of activation associations

Here we conduct an analysis to provide provisional evidence on whether the region of right vmPFC uncovered by our main analysis might be functionally connected with other brain regions during low ambivalence trials. The vmPFC region in question showed significant activations, aligned with the sympathetic response, and specifically during the offer period of

low ambivalence trials. We tested whether subjects with stronger activations in this region for this contrast also showed stronger activations in right (i.e. ipsilateral) amygdala (rAMY), dorsolateral prefrontal cortex (dlPFC) and anterior cingulate (ACC) for this contrast. These regions were defined by the Harvard-Oxford cortical structural probabilistic atlas (https://fsl.fmrib.ox.ac.uk/fsl/fslwiki/Atlases) and selected given their broad involvement in important cognitive mechanisms for the approach-avoidance paradigm, such as threat detection, conflict monitoring, value and cognitive control. We also ran this analysis for the direct low vs high ambivalence contrast, i.e., not modulated by the sympathetic response. For each contrast, we fitted three Bayesian regression models:

$$vmPFC(A) \sim a_{rAMY}(A) + \beta_{rAMY}(A) * X_{rAMY}(A) + \varepsilon_{rAMY}$$
$$vmPFC(A) \sim a_{dlPFC}(A) + \beta_{dlPFC}(A) * X_{dlPFC}(A) + \varepsilon_{dlPFC}$$
$$vmPFC(A) \sim a_{ACC}(A) + \beta_{ACC}(A) * X_{ACC}(A) + \varepsilon_{ACC}$$

(A) covers the two-by-two combination of time period (offer, commit) and action (accept, reject). The intercept (a_{rAMY} , a_{dlPFC} and a_{ACC}) and regression coefficient (β_{rAMY} , β_{dlPFC} and β_{ACC}) in each model were therefore fitted as four element vectors, mapping onto four separate regressors to estimate four separate vectors of observed data. Error was a single vector in each case. The intercept and regression coefficient vectors were assigned normal priors ($\mu=0,\sigma=1000$) while error was assigned a half-normal prior ($\sigma=1000$). Parameters were then updated by the observed data using Bayesian Markov chain Monte Carlo (MCMC), with posterior distributions sampled with No U-Turn sampling (NUTS), using PyMC3 (Salvatier et al. 2016) in Python 3.8. Posterior distributions were sampled across 4 chains of 10000 samples (40000 total), with an additional initial 10000 samples per chain (40000 total) discarded after tuning the sampler's step-size (80000 samples combined). We considered strong evidence of a significant association if 94% of the highest density interval (HDI95%) of the posterior of the relevant regression coefficient did not subtend zero.

Results of these models are summarized in figure S2. Note that regressors not showing significance are whited out of both matrix depictions. We can observe that overall associations were stronger for the relevant conditions for the basic ambivalence contrast (coefficient intensities ranging from 13.9 to 31.3) compared to the sympathetic contrast (5.83 to 9.78). However we also observe significant associations in more conditions (11/12 vs 8/12). Though a speculative analysis, it's noteworthy that the only association not significant for the sympathetic contrast is that between vmPFC and rAMY during the offer period, which is the condition we see the cluster reach significance in the primary analysis.

Contrast estimates in other ROIs

Here we report activations of our twelve contrasts in bilateral anterior cingulate cortex, paracingulate gyrus and subgenual cortex, and separately for right and left insula. Supplementary Table 1 summarizes the results. For each region, and for each contrast (I-XII; see methods section) we report the mean comparison of parameter estimates (fsl - cope) in that region in addition to the mean expected variance of contrasts (fsl - varcope). These regions were defined by the Harvard-Oxford cortical structural probabilistic atlas (https://fsl.fmrib.ox.ac.uk/fsl/fslwiki/Atlases) and selected given their involvement with either sympathetic activity (ACC, insula) or confidence/ambivalence (anterior medial PFC regions). Data are intended to be exploratory and illustrative and were not formally tested for significance.

Bayesian parameter of ambivalence-sympathetic association

Here we conduct an additional Bayesian analysis to assess the certainty of the regression parameter observed between ambivalence and sympathetic state (see *Behavior-sympathetic results)*. The benefit of this approach is that by using a wide uninformative prior (centred on 0), this model is more robust against false-positives (absent an effect, the sampler should converge on its starting point (0)). The model included only a parameter for ambivalence (to remove any effect of mediation from other parameters) and a separate intercept for each subject (analogous to a random intercept model).:

$$symp\ state(r) = z(n) + \beta * ambivalence(r) + \varepsilon$$

Where $r = run,\ n = subject$

The intercept and regression coefficient were assigned normal priors ($\mu=0, \sigma=100$) while error was assigned a half-normal prior ($\sigma=5$). Parameters were then updated by the observed data using Bayesian Markov chain Monte Carlo (MCMC), with posterior distributions sampled with No U-Turn sampling (NUTS), using PyMC3 (Salvatier et al. 2016) in Python 3.8. Posterior distributions were sampled across 4 chains of 1000 samples (4000 total), with an additional initial 10000 samples per chain (4000 total) discarded after tuning the sampler's step-size (8000 samples combined). The posterior for the *ambivalence-sympathetic association* had a mean of -0.340 and standard deviation of 0.203. The posterior was on the margin of showing strong evidence that the parameter is negative: 95.5% of posterior was below 0 and the highest density interval ranged from -0.722 to 0.044. This posterior could be used in a follow up study and serve as a Gaussian prior (i.e., $\mu = -0.340$; $\sigma = 0.203$) on this parameter estimate.

Supplementary references

Salvatier, J., Wiecki, T. V., & Fonnesbeck, C. (2016). Probabilistic programming in Python using PyMC3. *PeerJ Computer Science*, *2*, e55.
