# Supplementary figures and images for: Ventromedial Prefrontal Cortex Activity and Sympathetic Allostasis During Value-Based Ambivalence
Source: Front Behav Neurosci. 2021 Feb 22;15:615796. doi: 10.3389/fnbeh.2021.615796 (PMC7937876; doi:10.3389/fnbeh.2021.615796)

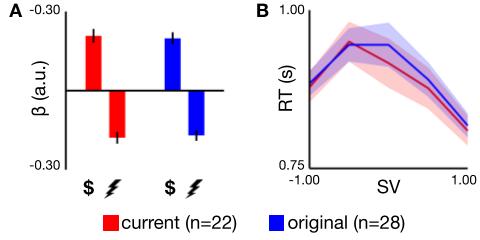

Supplement: Supplementary file 3 [file Image_1.JPEG]

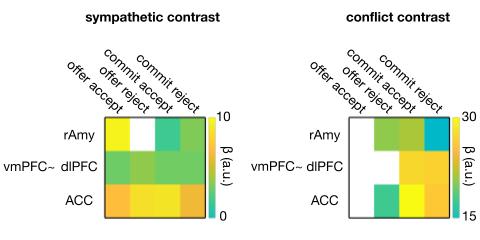

Supplement: Supplementary file 4 [file Image_2.JPEG]
